# Shared Autonomic Pathways Connect Bone Marrow and Peripheral Adipose Tissues Across the Central Neuraxis

**DOI:** 10.3389/fendo.2019.00668

**Published:** 2019-09-27

**Authors:** Natalie K. Y. Wee, Madelyn R. Lorenz, Yusuf Bekirov, Mark F. Jacquin, Erica L. Scheller

**Affiliations:** ^1^Division of Bone and Mineral Diseases, Department of Medicine, Washington University School of Medicine, St. Louis, MO, United States; ^2^Department of Reconstructive Sciences, UConn Health, Farmington, CT, United States; ^3^Department of Neurology, Washington University School of Medicine, St. Louis, MO, United States; ^4^Department of Cell Biology and Physiology, Washington University School of Medicine, St. Louis, MO, United States

**Keywords:** bone marrow adipose tissue, fat, brain-bone interactions, pseudorabies virus, viral tract tracing, energy metabolism, sympathetic nerve, autonomic nervous system

## Abstract

Bone marrow adipose tissue (BMAT) is increased in both obesity and anorexia. This is unique relative to white adipose tissue (WAT), which is generally more attuned to metabolic demand. It suggests that there may be regulatory pathways that are common to both BMAT and WAT and also those that are specific to BMAT alone. The central nervous system (CNS) is a key mediator of adipose tissue function through sympathetic adrenergic neurons. Thus, we hypothesized that central autonomic pathways may be involved in BMAT regulation. To test this, we first quantified the innervation of BMAT by tyrosine hydroxylase (TH) positive nerves within the metaphysis and diaphysis of the tibia of B6 and C3H mice. We found that many of the TH+ axons were concentrated around central blood vessels in the bone marrow. However, there were also areas of free nerve endings which terminated in regions of BMAT adipocytes. Overall, the proportion of nerve-associated BMAT adipocytes increased from proximal to distal along the length of the tibia (from ~3–5 to ~14–24%), regardless of mouse strain. To identify the central pathways involved in BMAT innervation and compare to peripheral WAT, we then performed retrograde viral tract tracing with an attenuated pseudorabies virus (PRV) to infect efferent nerves from the tibial metaphysis (inclusive of BMAT) and inguinal WAT (iWAT) of C3H mice. PRV positive neurons were identified consistently from both injection sites in the intermediolateral horn of the spinal cord, reticular formation, rostroventral medulla, solitary tract, periaqueductal gray, locus coeruleus, subcoeruleus, Barrington's nucleus, and hypothalamus. We also observed dual-PRV infected neurons within the majority of these regions. Similar tracings were observed in pons, midbrain, and hypothalamic regions from B6 femur and tibia, demonstrating that these results persist across mouse strains and between skeletal sites. Altogether, this is the first quantitative report of BMAT autonomic innervation and reveals common central neuroanatomic pathways, including putative “command” neurons, involved in coordinating multiple aspects of sympathetic output and facilitation of parallel processing between bone marrow/BMAT and peripheral adipose tissue.

## Introduction

Within the peripheral nervous system, sympathetic adrenergic signals are transmitted by several distinct sets of ganglia, which regulate regions in the head, trunk, viscera, and limbs. Common higher order processing centers are needed to ensure rapid, precise coordination of whole-body responses such as changes in vascular tone and energy metabolism. Consistent with this, the central nervous system (CNS) is recognized as a key mediator of peripheral adipose tissue function (1–7). The bone marrow is also an important site of peripheral adiposity with evidence for unique regulation and function [reviewed in (8)]. However, to date, very little is known about the neural control of bone marrow adipose tissue (BMAT) or its relationship to other adipose tissue depots across the central neuraxis.

The existence and prevalence of sympathetic neurons within the skeleton and bone marrow is well established (9–12). Ducy et al. first functionally demonstrated that central leptin administration reduced bone mass (13). This was later followed by other studies demonstrating that this effect was mediated via sympathetic nerves and modulation of β-adrenergic signaling (14, 15). Centrally, key neuropeptides associated primarily with the hypothalamus (e.g., NPY, CART, AgRP, POMC) have also been implicated in regulating bone homeostasis [reviewed in (16)]. Despite current work linking both the hypothalamus and sympathetic nerves to modulation of the bone microenvironment, the central regulatory regions influencing the skeleton are still relatively undefined.

We hypothesized that shared central neural pathways, relative to white adipose tissue (WAT), may be involved in BMAT regulation. To test this hypothesis, we performed viral transneuronal tract tracing from bone marrow and inguinal WAT. Viral tract tracing is a tool used to identify neural circuits. In particular, attenuated pseudorabies virus (PRV) recombinants such as the PRV-Bartha strain are well-established tracers that can be used for multi-synaptic directional tracing (17–19). After local PRV injection, all exposed viral axons within the site are infected. The virus then traffics to the cell body, replicates, and spreads across retrograde efferent synapses. This facilitates multi-synaptic tracing through the spinal cord and CNS. Whilst sensory cell bodies can be infected with PRV, they will not sort viral particles into central axons across afferent synapses and thus, viral transmission terminates in these cells. These properties make PRV tracers ideal for identifying and mapping efferent pathways from peripheral tissues, inclusive of those within the sympathetic nervous system (SNS).

Several previous reports have used tracing techniques to begin to map the higher order autonomic networks that regulate bone, adipose tissues, and other organs (4, 5, 20–27). However, shared regulatory regions between bone marrow/BMAT and peripheral WAT have not been identified. Thus, in this study, we first determined the proportion of BMAT adipocytes that are innervated by the SNS in C3H/HeJ (C3H) and C57BL/6J (B6) mice. Then, we used PRV to trace efferent neuroanatomical circuits from both tibial bone marrow (inclusive of BMAT) and inguinal WAT of C3H animals. Tracing from B6 femur/tibia was used as a control to examine strain- and skeletal site-specificity. To accomplish this, we used replication competent, isogenic, attenuated strains of the PRV virus (PRV-Bartha) in which the gG locus had been replaced with a fluorescent reporter (28).

## Methods

### Mice

The Institutional Animal Care and Use Committee (IACUC) at Washington University in St. Louis approved all procedures, and these experiments were performed in AAALAC accredited facilities. For all experiments, C3H/HeJ (C3H, Stock:000659) and C57BL/6J (B6, Stock:000664) mice were obtained from Jackson Labs; mice were acclimatized for 1 week prior to experiments. Mice were housed on a 12-h light/dark cycle at 70 ± 2 degrees Fahrenheit and fed standard chow (LabDiet® 5053). Relative to B6 mice, C3H mice are known to have a significant expansion of BMAT in the proximal tibia by 12-weeks of age (29).

### Retrograde Viral Tract Tracing

**Figure 1:**
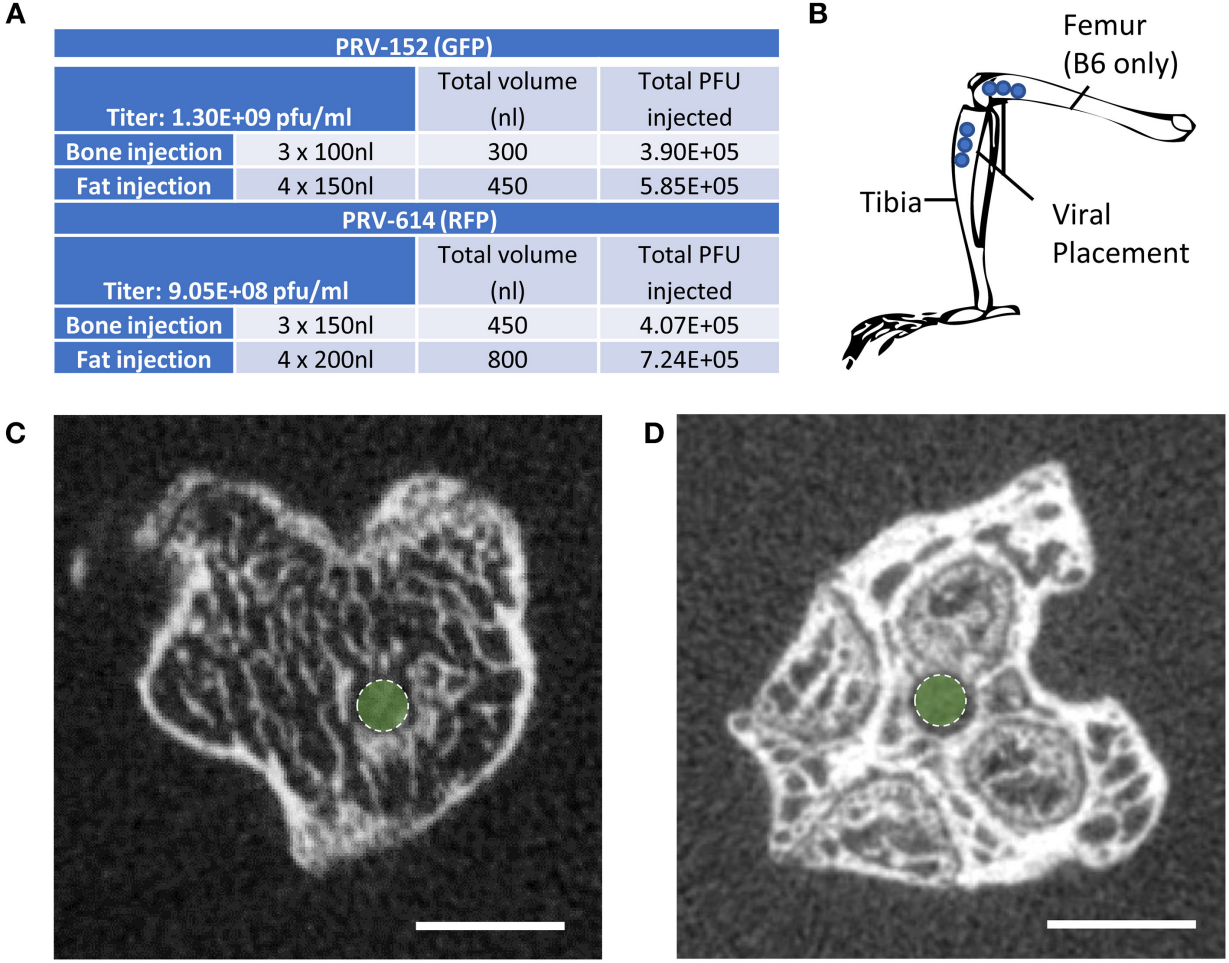
Virus placement and weight loss. **(A)** Viral titers and injection volumes. **(B)** Virus placement in the tibia at depths of 2.5, 3.0, and 3.5 mm from the top of the tibia or femur. Access to the tibial bone marrow was gained at the knee between the medial and lateral tibial condyles (B6 and C3H mice). Access to the femoral bone marrow was also via the joint surface (B6 mice only). **(C)** Representative computed tomography image of the needle tract into the tibial metaphysis (green dot). A pulled glass microinjector was used to minimize any disturbance to the surrounding bone and marrow. **(D)** Representative computed tomography of the needle tract into the femur (green dot).

Mice at 12-weeks of age underwent surgery and were euthanized for analysis 5–6 days after viral infection. This timing is sufficient to allow for retrograde transsynaptic transport of virus through 3 synaptic relays, up to 4 orders of neurons (1). Two isogenic pseudorabies retrograde tracing viruses were obtained from the NIH Center for Neuroanatomy with Neurotropic Viruses (CNNV): PRV-152 (30, 31) and PRV-614 (28). The PRV-152 virus has EGFP as the reporter, while mRFP is the reporter for PRV-614. C3H: Half the mice received PRV-152 into the tibia and PRV-614 into iWAT; whilst the other half received PRV-614 into the tibia and PRV-152 into iWAT. B6: Half the mice received PRV-152 into the proximal tibia and PRV-614 into distal femur; whilst the other half received PRV-614 into tibia and PRV-152 into femur. Figure 1A lists the viral loads injected at each site (fat vs. bone). A drilled bone defect model was used to place virus directly in bone using a pulled glass needle, microinjector, and stereotaxic apparatus to deliver 0.10 μL (PRV-152) or 0.15 μL (PRV-614) of viral solution at depths of 2.5, 3, and 3.5 mm from the top of the bone including the cartilage (Figures 1B–D). The injection site was sealed with bone wax to prevent leakage of the virus. For iWAT injections, 0.15 μL (PRV-152) or 0.20 μL (PRV-614) of virus was injected using a pulled glass needle at each of four sites along the length of the iWAT. All mice were given Buprenorphine SR (ZooPharm, 1.0 mg/kg) and monitored daily post-surgery until euthanasia. In our hands, 25% of injected C3H mice and 36% of injected B6 mice did not display evidence of infection in the brain. This could be due to failed ejection of the viral solution from the microinjector tip, failed viral infection, replication and/or spread, or mis-injection into the circulation. Previous work has shown that IV injection of PRV does not cause central neuronal infection (32).

### Tissue Collection

Mice were sedated with Ketamine/Xylazine and perfused through the left ventricle with 25 mL of phosphate buffered solution (PBS) followed by 25 mL 4% paraformaldehyde (PFA) at a rate of 5.0 mL per minute using a peristaltic pump. Tissues were post-fixed overnight in 4% PFA and then placed in PBS for storage or processed and analyzed as described below.

### MicroCT

Post-fixation, bones were embedded in 2% agarose gel. The proximal ends of the tibiae were scanned at 20 μm voxel resolution using a Scanco μCT 40 (Scanco Medical AG) calibrated using a hydroxyapatite phantom. Scans were used to verify placement of the needle into the bone (Figures 1C,D).

### Immunostaining and Analysis

#### Tibia

**Figure 2:**
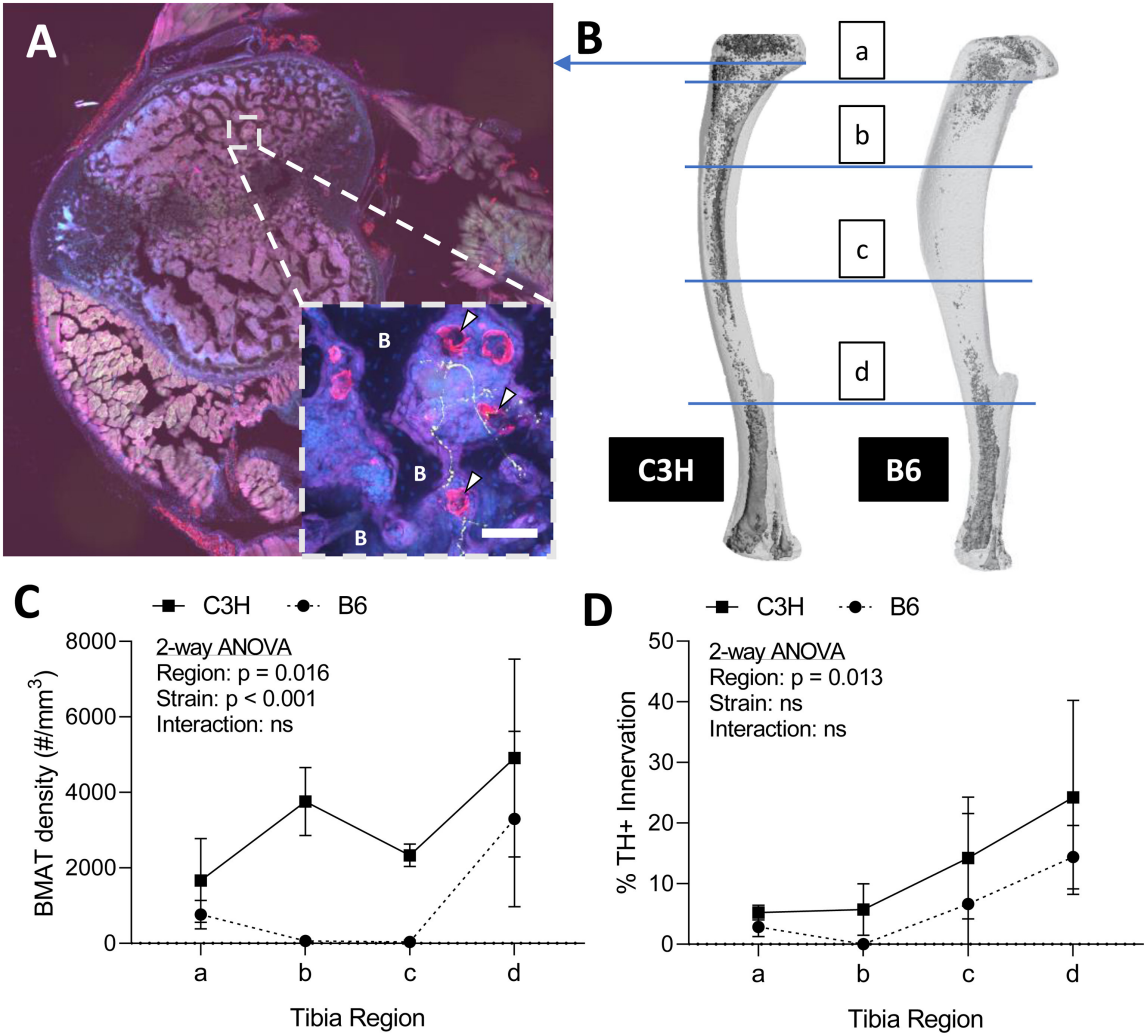
Innervation of BMAT adipocytes by sympathetic autonomic nerves. **(A)** Cross-section of the proximal tibial metaphysis with immunohistochemical stains for adipocytes (perilipin, red) and sympathetic adrenergic nerves (tyrosine hydroxylase, green) overlaid with dapi (blue). In some regions, perilipin+ adipocytes are observed immediately adjacent to TH+ axons (~1 μm in diameter, fibrous/branching, representative inset, white arrowheads). B, bone. Scale bar = 50 μm. **(B)** Representative distribution of bone marrow adipose tissue (BMAT) in a C3H and B6 male, 13-week old tibiae. Osmium stain and CT reconstruction. Immunohistochemical sections along the length of the tibia were analyzed for adiposity and TH+ innervation as indicated. **(C)** The number of perilipin positive adipocytes in a full tibial cross-section (see example in **A**) was counted in each region as indicated in **B** and normalized to the volume of the bone marrow. As previously reported Scheller et al. (29), C3H mice had increased density of BMAT adipocytes along the length of the tibia relative to B6 animals (2-way ANOVA). **(D)** In the same regions, the number of adipocytes <5 μm from a TH+ nerve were counted and expressed relative to the total number of adipocytes within the section [“% BMAT Innervation (TH+)”]. Despite differences in adipocyte density, the proportion of BMAT adipocytes that were adjacent to a TH+ axon remained relatively consistent between strains. In addition, the % of innervated BMAT adipocytes was highest in distal regions of the tibia of both B6 and C3H mice. *N* = 3, C3H; *N* = 5, B6. 2-way repeated measures ANOVA. Graphed as mean ± SD.

To assess skeletal innervation, whole tibiae from 13-week old C3H and B6 male mice were sectioned transversely at 50 μm along the length of the bone. Tissues were processed in 30% sucrose and embedded in Tissue-Plus OCT compound (Fisher Scientific, Hampton, New Hampshire, USA, 23-730-571) prior to cutting. Sections were blocked in 10% normal donkey serum (Sigma, St. Louis, MO, USA, D9663) in TNT buffer (0.1M Tris-HCl pH 7.4; 0.15 NaCl; 0.05% Tween-20). The sections were then incubated for 48-h with primary antibodies to tyrosine hydroxylase (TH) and perilipin (Supplemental Table 1). Following three rinses in TNT buffer, primary antibody staining was visualized using fluorescently-tagged secondary antibodies. The sections were rinsed again with TNT buffer and incubated in DAPI (1:1,000 dilution; Sigma, St. Louis, MO, USA, D9542) for 5-min before mounting with Fluoromount-G (ThermoFisher Scientific, Waltham, Massachusetts, USA, 00-4958-02). Tiled sections were imaged at 10X on a Nikon spinning disk confocal microscope. Images were reconstructed and analyzed in ImageJ/FIJI (33). The number of perilipin positive adipocytes was counted manually in each section and the proportion adjacent to a TH+ axon was recorded (<5 μm spacing). It is important to note that only TH+ structures the bone marrow with a size and morphology that was consistent with an autonomic axon fiber were considered in our analysis (size ~1 μm in diameter, fibrous/branching, no nuclei—see Figure 2A as an example).

#### Spinal Cord

After perfusion, processing in 30% sucrose, and embedding in Tissue-Plus OCT compound, whole embedded spines were stored at −80°C until sectioning. The entire spinal cord was sectioned at 50 μm on a cryostat (Leica CM1850) and then collected onto Superfrost charged slides (Fisher Scientific, Hampton, New Hampshire, USA, 12-550-15). Every 8th section was stained. The sections were thawed at room temperature for 10-min, prior to immunostaining. Sections were first blocked in 10% normal donkey in TNT buffer. The sections were then incubated for 48-h in primary antibodies against RFP and GFP (Supplemental Table 1). Following three rinses in TNT buffer, primary antibody staining was visualized using fluorescently-tagged secondary antibodies. The sections were rinsed again with TNT buffer and incubated in DAPI for 5 min before mounting with Fluoromount-G. The sections were imaged with a Hamamatsu 2.0-HT NanoZoomer at 20x magnification. Spinal cord sections were analyzed using the Allen Spinal Cord Atlas as a reference database (34).

#### Brain

Dry ice (solid CO_2_) was crushed to make a powder; each brain was rolled in the CO_2_ and then left to freeze solid on dry ice. Each sample was mounted using Tissue-Plus OCT compound to a H/I Cryo-Histomat MK-3 (Hacker Instruments, Winnsboro, South Carolina, USA) and cut into slices of 30 μm thickness. Sections were split into six series and stored in cryoprotectant solution (0.2M phosphate buffer pH 7.4, ethylene glycol, sucrose) until stained. Sections were washed in TNT buffer and blocked in 0.1M PBS with 0.05% Tween 20 and 5% normal donkey serum. Primary antibodies to GFP, RFP, and/or TH were diluted in 0.1M PBS with 0.05% Tween 20 and 1% normal donkey serum and were incubated overnight at 4°C (Supplemental Table 1). Following rinsing in TNT buffer, sections were incubated with secondary antibody for 1-h at room temperature (Supplemental Table 1). Sections were then incubated with DAPI (1:1,000) and then arranged onto slides and coverslipped with Fluoromount-G. Imaging was performed with a Hamamatsu 2.0-HT NanoZoomer at 20x magnification. Sections spanning the entire brain were matched to images in The Mouse Brain in Stereotaxic Coordinates Brain by Paxinos and Franklin (35) in order to identify traced central sites. For figure generation, a subset of sections were imaged at 10X magnification on a Leica confocal microscope.

#### Statistics

Statistics were performed in GraphPad Prism®. Statistical tests are indicated in the figure legends. An unpaired *t-*test was used to assess differences in body mass.

## Results

### Sympathetic Adrenergic Innervation of Tibial BMAT Adipocytes in C3H and B6 Mice

Sympathetic adrenergic axons in bone and adipose tissues are rich in TH+ varicosities and terminate as free nerve endings (9, 36, 37). The proximity of the axon facilitates diffusion of neurotransmitters and subsequent actions on surrounding target cells, such as adipocytes. Thus, we performed immunohistochemical analysis of TH+ adrenergic nerve fibers and quantified their relationship to perilipin positive BMAT adipocytes at four sites along the length of the tibia. Consistent with previous reports (9, 38), we found that many of the TH+ axons were concentrated around central blood vessels in the bone marrow. However, there were also sparse areas of free nerve endings, particularly in the proximal metaphysis, which terminated in regions of BMAT adipocytes (Figure 2A). The relationship of BMAT adipocytes with TH+ axons was further explored by quantifying the percent of BMAT adipocytes that were directly adjacent to an axon (<5 μm away) along the length of the tibia in both C3H and B6 mice (Figures 2B–D). These included all adipocytes along the length of the neuron, not just at the axon terminal. As expected, the tibial adipocyte density was significantly greater in C3H mice relative to B6, particularly within the diaphysis (Figure 2C). However, despite this difference in density, the proportion of innervated BMAT adipocytes was comparable between strains and increased gradually from proximal to distal along the length of the tibia (Figure 2D). Specifically, from the proximal metaphysis to the region of the tibia/fibula junction, innervation increased from 5.2 ± 1.2% to 24.2 ± 16.0% in the C3H mice and 2.9 ± 1.4% to 14.4 ± 4.5% in B6 (Figure 2D).

### PRV Tracing Identifies Central Pathways Mediating Efferent Innervation of Bone Marrow (Inclusive of BMAT) and iWAT

#### Infection Rate and Viral Characteristics

Male C3H mice at 12-weeks of age were injected with isogenic PRV viruses as detailed in Figures 1A,B. Half received PRV-152 (EGFP) into tibia and PRV-614 (mRFP) into iWAT, and the other half had PRV-614 (mRFP) into tibia and PRV-152 (EGFP) into iWAT. Though not uniformly reported or discussed in previous publications, PRV infection causes weight loss, lethargy, and eventually death in experimental animals (5, 19). C3H animals that were positively infected with one or both viruses demonstrated weight loss of 15 ± 1.6%, whilst mice negative for viral infection lost <3.9 ± 0.6% body weight (*p* = 0.003). At the end of the experiment, 75% of mice demonstrated positive infection with at least one virus that had progressed to the brain. Upon μCT verification of needle placement, one mouse was excluded due to needle perforation through the tibial cortical bone. The results from the remaining animals are described below.

### Spinal Cord

**Figure 3:**
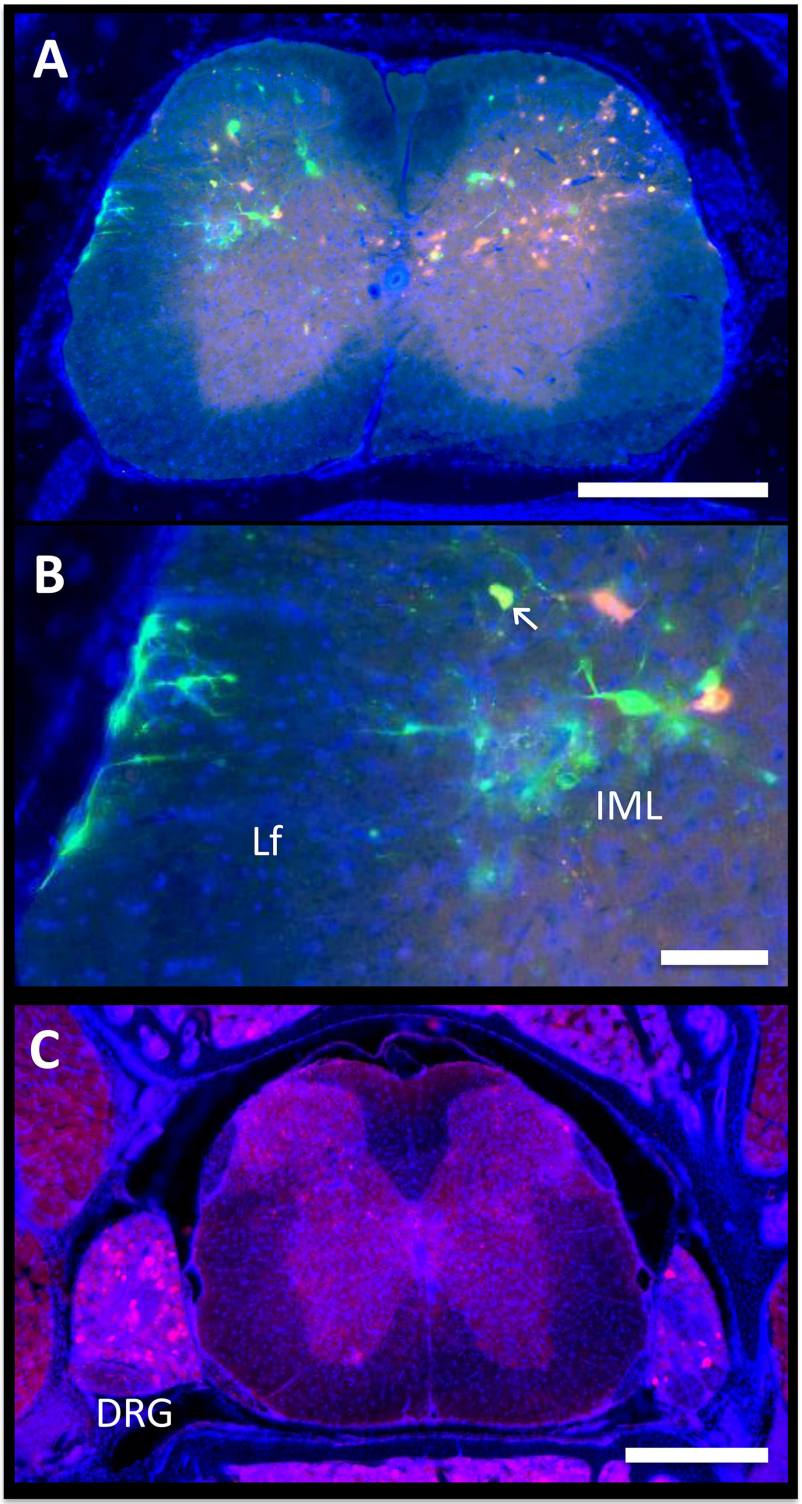
Spinal cord. The axon of the most distal neuron within bone or fat, also known as the postganglionic autonomic neuron, connects to its cell body in the sympathetic chain ganglia. Within the ganglion, this cell body synapses with a new neuron, the sympathetic preganglionic neuron (SPN). The cell bodies of the SPNs are located in the spinal cord in the intermediolateral (IML) nuclei. Spinal cord neurons are then controlled by supraspinal circuits from the brain, which send target axons to synapse on SPNs through descending pathways. These are ultimately responsible for the coordinated regulation of neuro-skeletal and neuro-adipose tissue interactions. **(A,B)** In the presented case, PRV-614 (RFP) was injected into the tibia, whilst PRV-152 (GFP) was injected into iWAT. Yellow neurons are neurons that have become infected with both viruses (arrow). Scale bar = 500 μm **(A)** and 100 μm **(B)**. Lf, lateral funiculus. **(C)** We observed positive cells within dorsal root ganglia (DRG) ipsilateral to the site of infection. In some cases, we also observed positive DRG cells on the contralateral side. However, spread to the brain through ascending spinal cord pathways was not noted. Representative image, PRV-614 (RFP) into iWAT. Scale bar = 500 μm.

Autonomic pathways consist of a two-neuron relay, which connects the tissue of interest to the spinal cord. In our experiments, the spinal cord was examined using serial thick frozen sections from the upper thoracic to the lower sacral regions. Labeling was observed to some extent at all levels of the thoracic, lumbar, and sacral spinal cord, except for the lowest sacral portions. Though a unilateral predominance was generally noted, the majority of the cases had progressed to a point where bilateral spread was evident. In all cases, cellular staining was notable in the sympathetic preganglionic neurons (SPNs) of the intermediolateral (IML) nuclei (Figure 3A). Connections from the IML were commonly present across the intercalated nucleus and labeling was also prominent within the central autonomic nucleus (lamina X). In the surrounding white matter, positive axons were noted to be crossing the lateral funiculus from the surface of the spinal cord (Figure 3B).

Positive axons were also observed in the lateral reticulospinal tract just outside of the IML, in laminae II, V, and VII, and occasionally in the ventral horn. In mice where dual labeling was present in the brain, the staining pattern was the same as described above. In addition, though the majority of labeled neurons in the spinal cord were of a single color/origin, a small subset of dual-labeled neurons was present.

Lastly, given our cross-sectional analysis paradigm, we were able to observe several of the dorsal root ganglia. Though the PRV virus used is only capable of crossing between neurons at retrograde synapses of efferent axons, it initially infects all axons within the target tissue, including sensory afferent neurons. Consistent with this, we observed positive staining in a subset of the dorsal root ganglia; infection within contralateral ganglia was also present in some cases (Figure 3C). However, further spread from these neurons through anterograde spinal cord pathways was not noted.

### Medulla, Pons, and Midbrain

**Figure 4:**
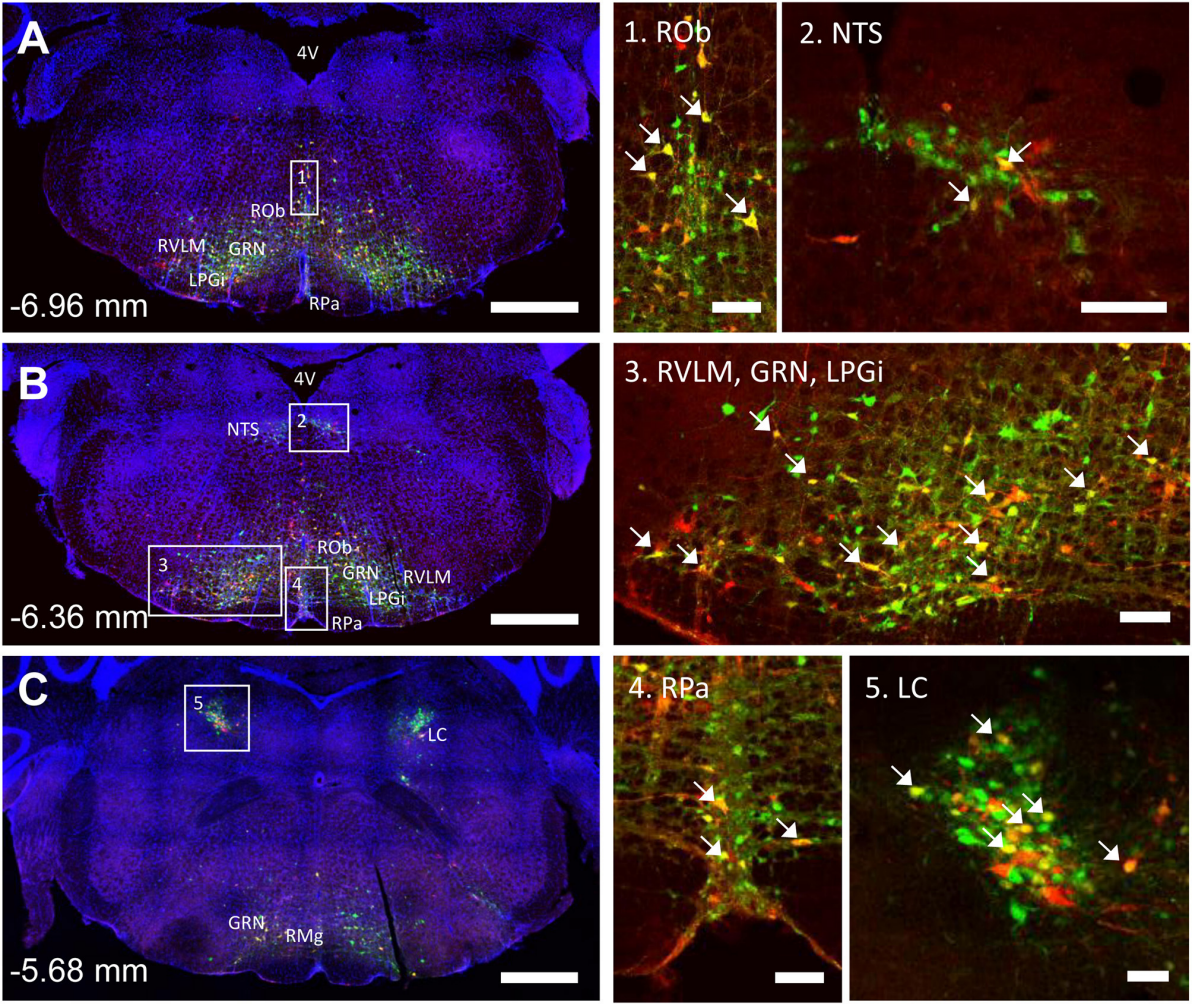
Co-infection of neurons from bone marrow/BMAT and iWAT in the medulla and pons. In the presented case, green neurons are traced from the tibia, whilst red neurons are from iWAT. Yellow neurons are those that have become infected with both viruses (white arrows). **(A,B)** Brainstem medulla, scale bar = 1 mm. **(C)** Pons, scale bar = 1 mm. Insets, scale = 100 μm: (1) Raphe obscurus (ROb), (2) nucleus of the solitary tract (NTS), (3) gigantocellular reticular nucleus (GRN), lateral paragigantocellular nucleus (LPGi) and rostral ventrolateral medulla (RVLM), (4) raphe pallidus (RPa), and (5) locus coeruleus (LC). RMg, raphe magnus.

**Figure 5:**
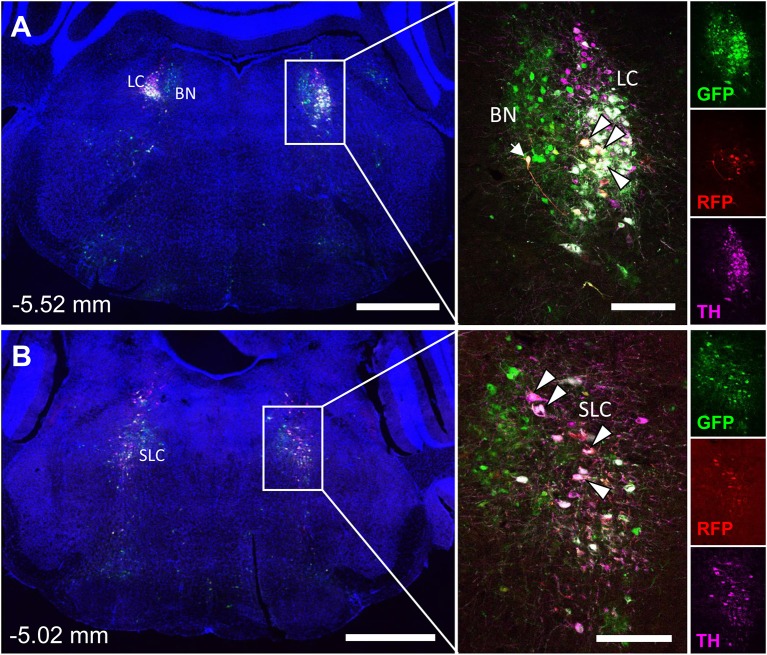
Tyrosine hydroxylase staining identifies co-infected ‘command neurons' in the locus coeruleus (LC), subcoeruleus (SLC), and Barrington's nucleus (BN). In the presented case, green neurons are traced from the tibia, whilst red neurons are from iWAT. A co-stain for tyrosine hydroxylase (TH) was used to define the boundaries of the locus coeruleus and subcoeruleus (LC and SLC, TH+) relative to Barrington's nucleus (BN, TH–). Yellow neurons are those that have become infected with both viruses without co-staining for tyrosine hydroxylase (TH) (white arrow). White neurons are those that are triple positive for GFP, RFP, and TH—indicating co-infection of a TH+ neuron within the LC or SLC as indicated (white arrowheads with black borders). **(A)** Overview of pons including the locus coeruleus (LC) and Barringtons nucleus (BN) at −5.52 mm from bregma. Scale bars: 1 mm and 200 μm (inset). **(B)** Overview of pons including the subcoeruleus (SLC) at −5.02 mm from bregma. Scale bars: 1 mm and 200 μm (inset). Individual channels for GFP (green), RFP (red), and TH (magenta) are shown for reference.

**Figure 6:**
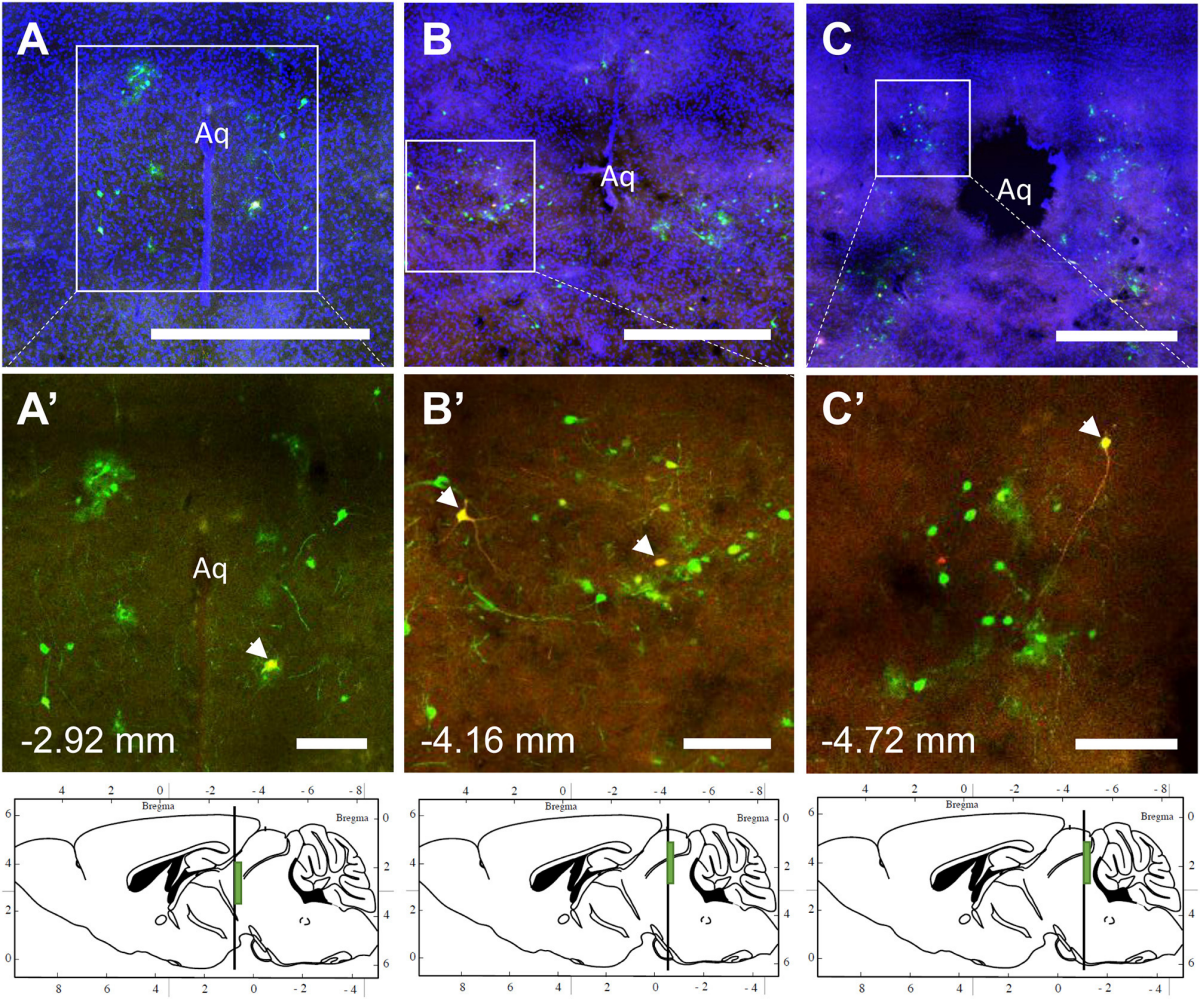
Co-infection of neurons from bone marrow/BMAT and iWAT in the periaqueductal gray (PAG). In the presented case, green neurons are traced from the tibia, whilst red neurons are from iWAT. Yellow neurons are those that have become infected with both viruses (white arrows). **(A–C)** Overview of tracing around the central aqueduct (Aq) at three regions including −2.92, −4.16, and −4.72 mm from bregma. Scale bar = 1 mm. **(A'–C')** Magnified insets showing individually traced neurons and those with co-infection from both sites (arrowheads). Scale bar = 200 μm. Green boxes on the sagittal sections denote the relative location of the displayed regions.

The spinal cord connects to the brainstem at the base of the skull, transitioning into the medulla. Consistent with previous studies (4, 5, 20–27), PRV infection occurred bilaterally within the brain, even though only the right tibia and right iWAT were injected. A summary of traced brain regions and their incidence is presented in Table 1. Within the medulla, we observed pronounced positive staining in the area postrema (AP), the nucleus of the solitary tract (NTS), including the medial and ventrolateral parts, and the reticular formation of the medulla, originating from both regions of BMAT and iWAT (Figures 4A,B; Table 1). Within the median reticular formation, the nucleus raphe obscurus (ROb), the raphe pallidus (RPa) and nucleus raphe magnus (RMg) were consistently traced with PRV (Figures 4A–C). Moderate staining was also consistently observed between samples in the gigantocellular reticular nucleus (GRN) and the lateral paragigantocellular nucleus (LPGi) (Figures 4A,B). Adjacent to the reticular formation, staining from bone marrow/BMAT and iWAT was notable in the rostroventral medulla (RVLM) (Figure 4B). In the pons, located between the medulla and the midbrain, tracing was prominent from both injection sites within the locus coeruleus (LC), subcoeruleus (SLC), and Barrington's nucleus (BN) (Figures 4C, 5A,B). As performed previously (22, 39), co-stains with TH were used to distinguish these three regions (Figure 5). In the midbrain, positive infection from bone marrow and iWAT was detected in the periaqueductal gray (PAG) (Figures 6A–C). Staining within the PAG was predominantly identified in the dorsomedial, lateral, and the ventrolateral areas.

**Table 1:** Traced brain regions from C3H tibial bone marrow [tibia, inclusive of bone marrow adipose tissue (BMAT)] and inguinal white adipose tissue (iWAT).

**Region of the brain**	**Abbreviation**	**Tibia** **(*N* = 5)**	**iWAT** **(*N* = 5)**	**Dual labeled neurons**
**MEDULLA AND RETICULAR FORMATION**				
Raphe obscurus nucleus	ROb	4	4	Yes
Raphe magnus nucleus	RMg	5	3	Yes
Raphe pallidus nucleus	RPa	5	4	Yes
Gigantocellular reticular nucleus	GRN	5	4	Yes
Lateral paragigantocellular nucleus	LPGi	4	4	Yes
Rostroventral medulla	RVLM	3	2	–
Nucleus of the solitary tract	NTS	5	4	Yes
Area postrema	AP	4	2	–
**PONS**				
Barrington's Nucleus	BN	5	5	Yes
Locus coeruleus	LC	5	5	Yes
Subcoeruleus nucleus	SLC	5	5	Yes
**MIDBRAIN**				
Dorsomedial periaqueductal gray	DMPAG	5	3	Yes
Lateral periaqueductal gray	LPAG	5	3	Yes
Ventrolateral periaqueductal gray	VLPAG	4	3	Yes
**HYPOTHALAMUS**				
Paraventricular hypothalamic nucleus, dorsal cap	PaDC	4	4	Yes
Paraventricular hypothalamic nucleus, lateral magnocellular part	PaLM	5	4	Yes
Paraventricular hypothalamic nucleus, medial magnocellular part	PaMM	5	3	Yes
Paraventricular hypothalamic nucleus, posterior part	PaMP	5	4	
Paraventricular hypothalamic nucleus, medial parvicellular part	PaPo	4	4	Yes
Lateral hypothalamus	LH	5	2	Yes
Posterior hypothalamic area	PH	4	1	–
Arcuate nucleus	Arc	3	1	–
Dorsomedial hypothalamus	DMH	2	2	Yes
Ventromedial hypothalamus	VMH	2	0	–
Suprachiasmatic nucleus	SCN	2	0	–
**OTHERS**				
Amygdala	Me	3	2	–
Pyriform cortex	Pir	2	2	–

In multi-labeled samples, dual infected neurons from both injection sites were present in the medullary reticular formation (ROb, RMg, RPa, GRN, LPGi) and NTS (Figure 4). We similarly observed a subset of pontine LC, SLC, and BN neurons that were co-infected with viruses originating from bone marrow and iWAT (Figure 5). Lastly, several dual traced neurons were present in the PAG (Figure 6).

### Hypothalamus and Forebrain

**Figure 7:**
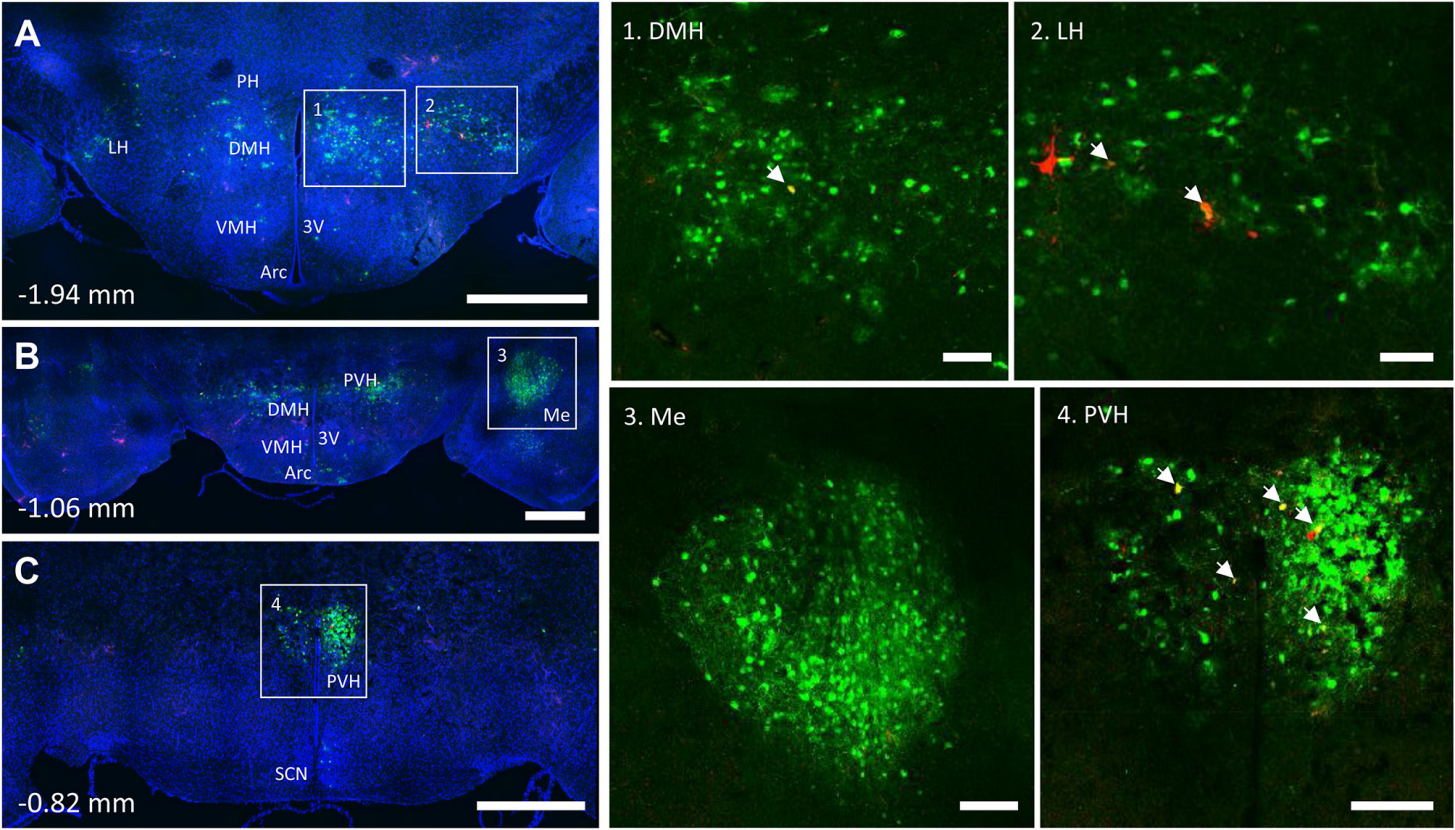
Co-infection of neurons from bone marrow/BMAT and iWAT in the hypothalamus. In the presented case, green neurons are traced from the tibia, whilst red neurons are from iWAT. Yellow neurons are those that have become infected with both viruses (white arrows). **(A)** Overview of hypothalamic tracing at −1.94 mm from bregma. Scale bar = 1 mm. **(B)** Overview of hypothalamic tracing, including amygdala, at −1.06 mm from bregma. Scale bar = 1 mm. **(C)** Medial portion of the paraventricular hypothalamus and suprachiasmatic nucleus at −0.82 mm from bregma. Scale bar = 1 mm. Insets: (1) dorsomedial hypothalamus (DMH), scale = 100 μm, (2) lateral hypothalamus (LH), scale = 100 μm, (3) amygdala (Me), scale = 200 μm and (4) paraventricular hypothalamus (PVH), scale = 200 μm. Regions identified include: third ventricle (3V), arcuate nucleus (Arc), dorsomedial hypothalamus (DMH), lateral hypothalamus (LH), amygdala (Me), posterior hypothalamus (PH), paraventricular hypothalamus (PVH), and suprachiasmatic nucleus (SCN).

PRV infection was prominent within the hypothalamus, most notably within the paraventricular hypothalamus (PVH) (Figure 7; Supplemental Figures 1, 2). Infection of the PVH was bilateral from both bone marrow and iWAT; however, there was typically a discernable difference with a greater number of neurons stained on one side than the other (Supplemental Figure 1). This may be due to the virus crossing the midline via interneurons and then proceeding up into the brain, thus viral infection may lag on contralateral side, leading to the observed difference. In dual positive-infected mice, we observed a substantial number of neurons arising from the tibial injection and a number of neurons arising from iWAT in both posterior and medial parts of the PVH (Figures 7A–C). Again, similar to regions of the medulla and pons, we also identified neurons that were co-infected with both viruses (Figure 7; Table 1). Other regions of the hypothalamus with positive PRV infection include the lateral hypothalamus (LH), posterior hypothalamic area (PH), arcuate nucleus (ARC), dorsomedial hypothalamus (DMH), ventromedial hypothalamus (VMH), and suprachiasmatic nucleus (SCN) (Figure 7; Table 1). Lastly, we found robust labeling of neurons in the amygdala in a subset of animals (Figure 7B; Supplemental Figure 3). In close proximity to the amygdala, PRV-infected neurons were also present in the pyriform cortex in a 2/5 tracings from bone marrow/BMAT and iWAT (Table 1; Supplemental Figure 3).

### PRV Tracing From B6 Femur and Tibia Mimics That Observed From C3H Mice

To examine the strain- and skeletal site-specificity of PRV, we traced to the brain from the proximal tibia and distal femur of a matched set of male 12-week-old, B6 mice. As above, some mice received PRV-152 (EGFP) into tibia and PRV-614 (mRFP) into femur while in others this was reversed. At the end of the experiment, needle placement was confirmed with μCT. Due to minor issues with tissue processing, the medulla and reticular formation could not be included in these analyses. However, in the pons, infection from the tibia and femur was observed in the BN, LC, and SLC (Table 2). Staining from both sites was also identified in the midbrain, specifically within the PAG (Table 2). As in C3H mice, the hypothalamus contained positive PRV infection predominantly in the PVH (Table 2; Supplemental Figure 4). Additional traced neurons were identified in the LH, PH, DMH, and SCN from a subset of animals (Table 2). Lastly, one of four cases from the tibia and two of three from the femur resulted in PRV infection in the amygdala (Table 2).

**Table 2:** Traced brain regions—B6 tibia and femur bone marrow (inclusive of BMAT).

**Region of the brain (from B6 mice)**	**Abbreviation**	**Tibia** **(*N* = 4)**	**Femur** **(*N* = 3)**
**PONS**			
Barrington's Nucleus	BN	4	2
Locus coeruleus	LC	4	3
Subcoeruleus nucleus	SLC	3	3
**MIDBRAIN**			
Dorsomedial periaqueductal gray	DMPAG	0	1
Lateral periaqueductal gray	LPAG	1	2
Ventrolateral periaqueductal gray	VLPAG	3	2
**HYPOTHALAMUS**			
Paraventricular hypothalamic nucleus, dorsal cap	PaDC	4	3
Paraventricular hypothalamic nucleus, lateral magnocellular part	PaLM	3	3
Paraventricular hypothalamic nucleus, medial magnocellular part	PaMM	3	3
Paraventricular hypothalamic nucleus, posterior part	PaMP	4	3
Paraventricular hypothalamic nucleus, medial parvicellular part	PaPo	4	3
Lateral hypothalamus	LH	2	2
Posterior hypothalamic area	PH	2	2
Arcuate nucleus	Arc	0	2
Dorsomedial hypothalamus	DMH	2	2
Ventromedial hypothalamus	VMH	0	2
Suprachiasmatic nucleus	SCN	2	2
**OTHERS**			
Amygdala	Me	1	2
Pyriform cortex	Pir	0	2

## Discussion

Shared innervation has been established between peripheral WAT and BAT adipose tissues (1–7). However, to date, our understanding of neural interactions with bone marrow/BMAT has been limited. We have previously demonstrated that cold exposure, a model of elevated sympathetic tone and catecholamine release, depleted BMAT from the proximal end of the tibia (29). More recently, it was shown that bone marrow adipocytes respond to isoproterenol (pan-adrenergic agonist—β1, β2, and β3) by increased phospho-hormone sensitive lipase, whilst the response following treatment with a β3 agonist, a major regulator of lipolysis in other adipose tissues, was present at a lower level (40). Together, this information suggests that there is likely some shared regulation of adipose tissues within the body but also that there may be subtle differences between BMAT and other adipose depots. Building on previous work, this study has been able to establish the presence of shared autonomic pathways between BMAT and iWAT. Dual labeled “command” neurons were noted regions such as the reticular formation, NTS, LC, PBN, SLC, BN, and hypothalamus—indicating that common neurons may be involved in the central regulation of both sites. Though the prevalence of dual infection reflects a wide range of variables including initial infection and viral trafficking, the overlap in our current study appears to range from 7 to 18% depending on the region (Figures 4–7). This is consistent with previous work on dual injections of PRV into different adipose depots which identified an incidence of 5–55% of dual-infected neurons, indicative of shared innervation between fat depots (41). The results provide the foundation for future studies to evaluate the functional roles of the identified central regulatory regions on BMAT, particularly within the contexts of shared regulation of WAT, bone, and bone marrow.

### Innervation of BMAT

The presence of sympathetic neurons in the skeleton is well established (9–12). Similarly, the innervation of WAT adipocytes is well-documented (42, 43). In WAT, EM studies demonstrate that ~5% of WAT adipocytes are immediately adjacent to a sympathetic nerve axon (43). By contrast, beige or brown adipocytes have an innervation rate that approaches 100%, often with multiple nerve fibers per cell (43, 44). Our results suggest that TH+ innervation of BMAT is more similar to what has been documented for WAT, with ~5–25% of the BMAT adipocytes located immediately adjacent to a TH+ axon (Figure 2). This helps to define when and where locally-released neurotransmitters have the potential to act directly on the cells. Conversely, it suggests that upwards of 70% of BMAT adipocytes are relatively disconnected from the local adrenergic nerve supply (though regulatory impulses could be communicated indirectly or by diffusion). In the metaphysis, TH+ axons were occasionally observed to branch and terminate in regions of BMAT. In the diaphysis, the trajectory of the axons was generally restricted to the arterial vasculature, with less branching. It is unknown whether positioning of a BMAT adipocyte near an axon along its length vs. at the termini impacts the ability of the neuron to act on the cell. In other organ systems, axons have been shown to release neurotransmitters along their entire length (45). More work is needed, however, based on this data it is clear that TH+ neurons are well-positioned to signal to a subset of BMAT cells.

### Shared Pathways—Vasoregulatory Responses

**Figure 8:**
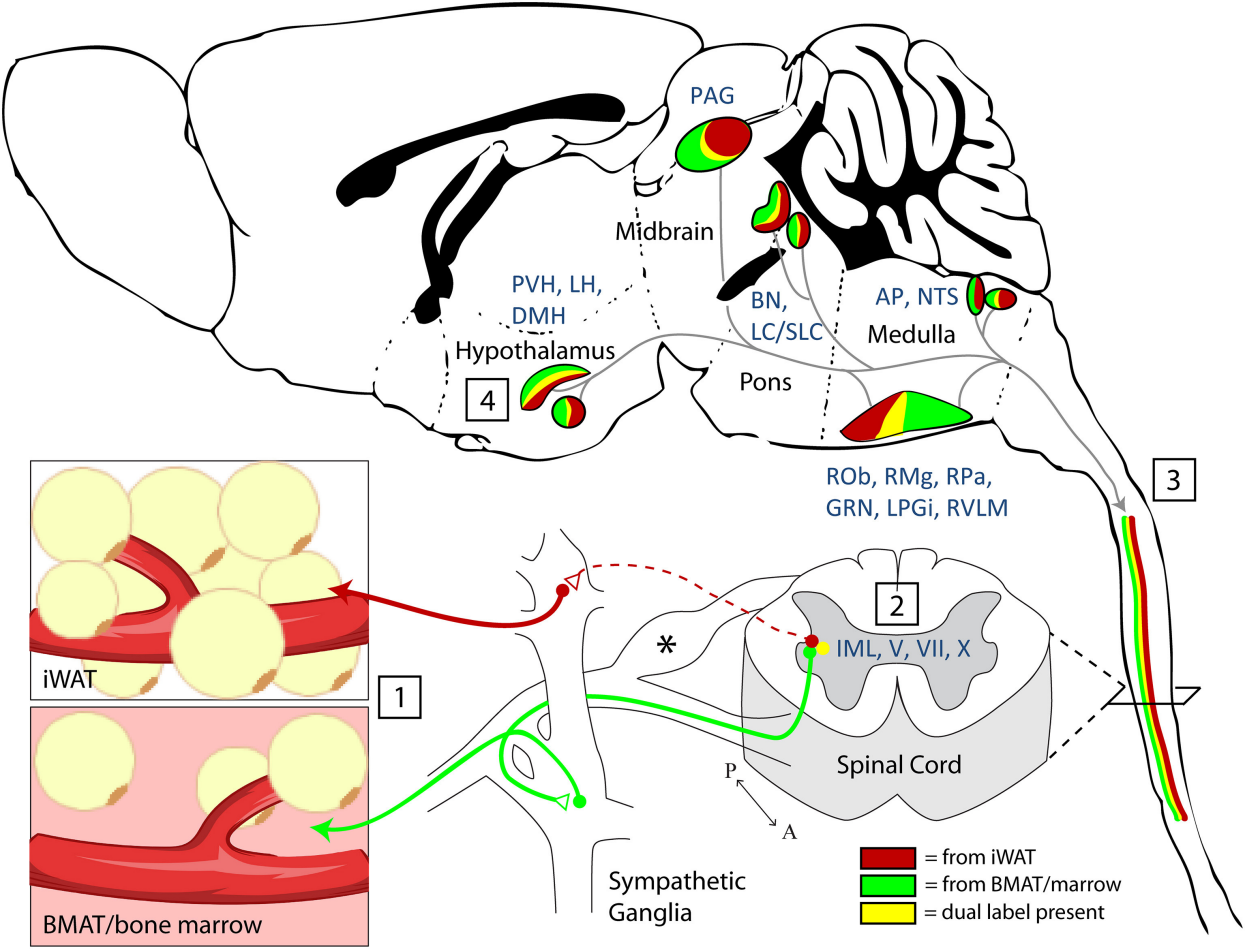
Summary and model. (1) Nerve endings were infected by PRV-bartha virus after local injection into inguinal white adipose tissue (iWAT) or regions of BMAT in the proximal tibia. (2) Infection of sympathetic post-ganglionic axons and their ganglionic cell bodies progressed to infect the sympathetic preganglionic neurons (SPNs) within the intermediolateral nucleus (IML) of the spinal cord. Infection within the spinal cord was also noted across the intercalated nucleus and in laminae V, VII, and X. ^*^Infection was also present in the dorsal root ganglia. This is due to the ability of PRV-Bartha to infect free endings of afferent neurons. However, after this, it is not able to traffic across afferent synapses toward the brain. (3) Viral tracing ascended through efferent pathways to central brain regions (4). The full list of traced regions is available in Tables 1, 2. Functionally, traced central regions are capable of coordinating autonomic signals through shared pathways, which descend to the target tissues. These regions have previously been implicated in regulation of vascular tone, lipolysis, and bone turnover.

A key strength of these experiments is our ability to examine the results within the context of the extensive range of previously published PRV tracing studies across most major organ systems. Upon doing so, several patterns emerge. First, there are multiple regions that have been traced in nearly all studies to date. This includes early infection in areas of the pontine and medullary reticular formation, RVLM, raphe nuclei, and PVH from organs including spleen (21, 22), kidney (23, 24), adrenal gland (25), BAT (5, 27), sympathetic ganglia (25), pancreas (26), lumbar muscle (46), and iWAT/eWAT (4). These structures represent a common neural circuit controlling sympathetic autonomic outflow to a diverse set of organs (Figure 8). One likely explanation for this is the need for coordinated, whole-body regulation of vascular tone by the autonomic nervous system (47). For example, functional studies in cats and primates demonstrate that stimulation of the PVH causes systemic vasopressor responses that are mediated by the descending autonomic vasomotor fibers on the surface of the spinal cord, which synapse on SPNs in the IML to signal to peripheral tissues (48). In our study, this is consistent with prominent labeling in regions including the PVH (Figure 7), the surface of the lateral funiculus of the spinal cord, the SPNs of the IML nucleus and the medially associated cord/SPN regions such as the intercalated nucleus and central autonomic area (lamina X) (Figure 3) (49). Similarly, the pre-sympathetic neurons of the RVLM, traced from both sites in this study, are a key source of excitatory inputs to the SPNs in the spinal cord that help to maintain baseline arterial pressure (Figure 4) (50).

Many of these areas have also been implicated in reflex autonomic control of vascular tone, a collection of diverse mechanisms by which the body integrates information from peripheral sensory inputs to subsequently coordinate autonomic responses. Within the spinal cord, for example, stimulation of sensory roots can influence the activity of autonomic SPNs (48). Locally, this may be explained by modulation of SPNs by autonomic interneurons within the dorsal horn (Figure 3) [discussed in (51, 52)]. To date, pre-sympathetic interneurons have been identified in laminae V, VII, and X. In our study, we also observed tracing in these laminae (Figure 3), emphasizing the potential for integration and modulation of sensory and sympathetic signals to iWAT/BMAT within the spinal cord (48). Integration also occurs within the brain. From this study, labeled central sites including the LC, NTS, RVLM, and medullary raphae (ROb, RMg, RPa) have the potential to integrate peripheral sensory inputs (e.g., somatosensory stimuli, peripheral chemoreceptors, arterial baroreceptors) with subsequent regulation of autonomic responses, including vascular tone (47, 50, 53–55). Thus, our work puts bone marrow/BMAT and iWAT into the same vasoregulatory network as organs including the spleen, kidney, adrenal gland, BAT, muscle and pancreas; however, future functional studies are needed to explore differences, if any, in the magnitude and timing of centrally-evoked responses.

### Shared Pathways—Energy Utilization and Lipolysis

Nerve endings in both WAT and bone marrow exist as free terminals and lack conventional synapses with surrounding cells [reviewed in (56)]. Thus, peripheral neurotransmission is mediated by bulk release and diffusion of signaling factors. The catecholamine norepinephrine has a well-established role in mediating SNS activity to peripheral tissues. Norepinephrine controls local SNS-mediated vasoregulation (57), SNS-mediated lipolysis (56, 58), and SNS-mediated thermogenesis/beiging (59). Though its expression is not restricted to neurons, there is also evidence that neuropeptide Y (NPY), another sympathetic neurotransmitter, can regulate peripheral adipose tissues both *via* central circuits and locally through direct actions on adipocytes and surrounding cells (60, 61). This emphasizes that those central pathways which cause bulk peripheral neurotransmitter release, as described above for vasoregulation, may also promote lipolysis and/or adipose tissue thermogenesis. Few studies have examined the effect of sympathetic neurotransmitters on BMAT directly and this is an area that could be the further explored in the future.

Central integration of peripheral inputs is similarly critical to ensure optimal autonomic contributions to energy partitioning. A key mediator of this relationship is the adipocyte-secreted hormone leptin. Our viral tracing demonstrated prominent labeling from iWAT and bone marrow/BMAT in leptin-responsive regions such as the PVH, ARC, DMH, VMH, and AP. In addition to known actions on food intake, leptin has been implicated in the central regulation of bone marrow and peripheral adipocytes. Studies examining leptin deficient models (*ob/ob*) have found increased BMAT (62, 63). Leptin treatment of *ob/ob* mice reduces BMAT number and size due to lipid mobilization and apoptosis whilst simultaneously increasing bone formation (63, 64). In addition, leptin delivery to the VMH of healthy rats for 5 days induces a significant depletion of peripheral fat pads and also BMAT (65); this functionally demonstrates that central regulatory regions such as the VMH, also identified in our study, can simultaneously influence both BMAT and peripheral adipose tissues.

### Regulation of Skeletal Homeostasis

While our focus is on adipose tissue comparisons, it should be noted that the bone injections would label nerves that interact with a heterogeneous population of cells: bone cells, bone marrow, and BMAT. Signaling in the VMH, for example, may link bone marrow with splenic innervation. Functionally, the VMH has been shown to suppress splenic lymphocyte activity (66) and natural killer cell cytotoxicity (67) demonstrating a clear role for sympathetic regulation of hematopoietic cells in spleen that may mirror what has been described for bone marrow (68).

In addition to hematopoiesis, for the past two decades, there have been numerous studies demonstrating the effects of central regulation on bone mass; interactions between the brain and bone mass have primarily focused on the hypothalamus (13, 14, 69). Consistent with this, we observed robust staining from bone to hypothalamic regions: LH, PH, ARC, DMH, VMH, and SCN. To date, the arcuate nucleus has a well-established role in regulating bone mass through AgRP/NPY neurons (70, 71) and more recently Kiss neurons (72). Interestingly, deletion of the estrogen receptor from Kiss1 neurons had sex-specific effects on bone mass with females having a striking 500% increase in cancellous bone mass in the distal femur, whilst males had normal bone mass in comparison to control WT mice (72). These studies, while focused on a single regulatory center of the brain, the arcuate nucleus, demonstrate the potential impact of modulating neuronal activity on the bone microenvironment. This same region is strongly linked to the regulation of energy homeostasis and the modulation of BAT and WAT, thus is a good candidate to further study to elucidate the effect of arcuate neurons on BMAT.

Similar to other adipose tissues, the bone microenvironment is also influenced by circulating cues such as leptin, which can influence sympathetic tone and also act directly on progenitor cells within the skeleton [reviewed in (73)]. Early work by Karsenty et al. demonstrated that leptin could inhibit bone formation via a central hypothalamic relay (13); this work predominantly examined the effects on vertebral bone mass, a site in the mouse that does not typically have many bone marrow adipocytes. The effects of leptin on bone have been complicated and been much debated; in *ob/ob* mice restoration of peripheral leptin signaling has an anabolic effect on bone mass (74), which opposes the central effects. This was also demonstrated when Prrx1-cre or Col3.6-cre was used to remove the leptin receptor; these mice had a significant increase in bone mass (75, 76).

In addition to the hypothalamus, the area postrema (AP) is another region that can respond to physiological factors as they enter the CNS and can influence autonomic control (77). The subpostrema, a V-shaped area, is located adjacent to the AP and on the upper limit of the commissural part of the NTS; it too is involved in autonomic regulation and provides bidirectional connections between area postrema and the NTS (78). Recently, Zhang et al. determined that Neuropeptide FF receptor 2 (Npffr2) signaling regulated NPY neuron activity in the arcuate nucleus and influenced BAT activity via the PVH (79). In addition to Npffr2 expression in the ARC, retrograde tracing from the ARC demonstrated that brainstem regions such as the area postrema, subpostrema, and NTS could provide input and regulate ARC neurons (79). Npffr2 deficient mice on a high fat diet show increased adiposity, reduced whole body energy expenditure, reduced UCP-1 protein in BAT and increases in cancellous bone mass (79). This study shows that brainstem neurons are able to influence the hypothalamus and regulate the bone microenvironment.

Other factors also implicated in energy homeostasis have been shown to influence the bone microenvironment *via* central signaling. Kajimura et al. (80) showed that adiponectin could influence sympathetic tone by regulating neurons within the locus coeruleus; leading to an inhibition of bone formation and increased bone resorption through RANKL (80). Notably, the central actions of adiponectin were apparent in older adiponectin deficient mice and differed from younger mice. Young adiponectin deficient mice had reduced bone mass, consistent with the direct effects of adiponectin to inhibit proliferation and induce apoptosis in osteoblasts (80). Similarly, hypocretin (also known as orexin) has been reported to reduce serum leptin levels *via* central signaling and reduce cancellous bone mass (81). Although sympathetic tone was not evaluated in that study, the changes in central orexin signaling were abolished in leptin deficient (*ob/ob*) mice suggesting that changes in leptin levels are affecting sympathetic tone (81). These studies highlight the notion that peptides can act through multiple pathways and thus, our study is a good tool for identifying potential regulatory regions that influence BMAT to assist with examining and targeting these central effects.

## Limitations

While we hypothesize that there may be unique sites that signal to the BMAT environment in a context-specific manner, our viral tract tracing is limited as it is only able to establish the presence of infection and co-infection *vs*. iWAT. For example, we identified infected neurons from bone marrow/BMAT in only 2 out of 5 cases of positive PRV infection in the VMH, but not when traced from iWAT (Table 1). However, previous work has shown that stimulation of the leptin-responsive neurons in the VMH significantly affects both WAT and BMAT adipose tissue depots (65). Thus, the absence of infection in neuronal tracing studies does not mean that a region is functionally unimportant. In addition, while we can focus and integrate information known about single neural sites, it is also important to consider that these central regulatory regions are connected and interact with each other, i.e., the arcuate nucleus sends projections to other regions within the hypothalamus (82), that subsequently project to other parts such as the locus coeruleus, solitary tract, and reticular formation. Thus, several regions may actually work together to regulate bone, bone marrow and BMAT. Lastly, though expression of TH has been widely used to characterize peripheral nerves within bone, bone marrow, and the periosteum (9, 11, 83, 84), it has the potential to be upregulated with nerve stimulation (85). Thus, it is possible that the proportion of TH+ nerve-associated BMAT adipocytes may be higher in settings of increased sympathetic tone.

## Prospectus and Conclusion

A large proportion of work into understanding the neural regulation of adipose tissue was originally performed in Siberian hamsters. Siberian hamsters (*Phodopus sungorus*) display large variations in body composition depending on the photoperiod they are exposed to: hamsters exposed to long days can have around 50% adiposity whilst exposure to shorter days leads to around 20% adiposity (86, 87). This connection between circadian rhythm and adiposity suggested a potential role for the CNS in regulating adipose tissue. While there are distinctive roles of the SNS in regulating brown adipose tissue (BAT) function in comparison to WAT, PRV retrograde tracing reveals that the central regulatory regions are similar between these peripheral adipose depots (5, 27). Consistent with this, we demonstrate that PRV tracing from bone marrow/BMAT identifies many of the same regions. In addition, we define a novel population of dual PRV-infected “command” neurons that are connected to both bone marrow/BMAT and iWAT. These neurons may coordinate multiple aspects of sympathetic output and facilitate parallel processing for local effects such as lipolysis, thermogenesis and vasoregulation (88–92). Moving forward, more work is needed, both at the level of the brain and locally within the bone marrow, to understand how and in what contexts neural impulses are necessary regulators of BMAT function.

## Data Availability Statement

The datasets generated for this study are available on request to the corresponding author.

## Ethics Statement

The Institutional Animal Care and Use Committee (IACUC) at Washington University in St. Louis approved all procedures, and these experiments were performed in AAALAC accredited facilities.

## Author Contributions

NW and ES conception of the work. NW, ML, YB, MJ, and ES acquisition and analysis of data, approved final copy of manuscript. NW, MJ, and ES interpretation of the data and drafting of the manuscript.

### Conflict of Interest

The authors declare that the research was conducted in the absence of any commercial or financial relationships that could be construed as a potential conflict of interest.

## Supplementary Material

Supplemental Figure 1 Paraventricular hypothalamus from all C3H mice injected with PRV into the tibia or iWAT. Medial portion of the paraventricular hypothalamus from each mouse injected with either PRV-152 (GFP) or PRV-614 (RFP) and the site of injection, tibia or iWAT. Scale bar: 1 mm.

Supplemental Figure 2 PRV infection from bone marrow/BMAT traces to various parts within paraventricular hypothalamus. PRV-152 (GFP) was injected into the tibia and the following sites showed PRV infection: **(A)** Suprachiasmatic nucleus (SCN) and paraventricular hypothalamic nuclei: dorsal cap (PaDC), lateral magnocellular part (PaLM), and medial magnocellular part (PaMM); **(B)** Posterior paraventricular hypothalamic nuclei: posterior part (PaMP) and medial parvicellular part (PaPo).

Supplemental Figure 3 Amygdala and pyriform cortex traced from tibia. **(A)** Overview of brain slice. **(B)** Amygdala. **(C)** Overview of pyriform cortex relative to amygdala. **(D)** Pyriform cortex.

Supplemental Figure 4 Paraventricular hypothalamus from all B6 mice injected with PRV into the tibia or iWAT. Medial portion of the paraventricular hypothalamus from each mouse injected with either PRV-152 (GFP) or PRV-614 (RFP) and the site of injection, tibia or iWAT. Scale bar: 1 mm.

Supplemental Table 1 Antibodies used for immunostaining.
