# Corrigendum: Shared Autonomic Pathways Connect Bone Marrow and Peripheral Adipose Tissues Across the Central Neuraxis

**DOI:** 10.3389/fendo.2019.00904

**Published:** 2020-01-22

**Authors:** Natalie K. Y. Wee, Madelyn R. Lorenz, Yusuf Bekirov, Mark F. Jacquin, Erica L. Scheller

**Affiliations:** ^1^Division of Bone and Mineral Diseases, Department of Medicine, Washington University School of Medicine, St. Louis, MO, United States; ^2^Department of Reconstructive Sciences, UConn Health, Farmington, CT, United States; ^3^Department of Neurology, Washington University School of Medicine, St. Louis, MO, United States; ^4^Department of Cell Biology and Physiology, Washington University School of Medicine, St. Louis, MO, United States

**Keywords:** bone marrow adipose tissue, fat, brain-bone interactions, pseudorabies virus, viral tract tracing, energy metabolism, sympathetic nerve, autonomic nervous system

There is an error in the **Funding** statement. The correct number for “U01-DR116317” is “U01-DK116317.”

The authors apologize for this error and state that this does not change the scientific conclusions of the article in any way. The original article has been updated.

