# Supplementary figures and images for: Shared Autonomic Pathways Connect Bone Marrow and Peripheral Adipose Tissues Across the Central Neuraxis
Source: Front Endocrinol (Lausanne). 2019 Sep 27;10:668. doi: 10.3389/fendo.2019.00668 (PMC6776593; doi:10.3389/fendo.2019.00668)

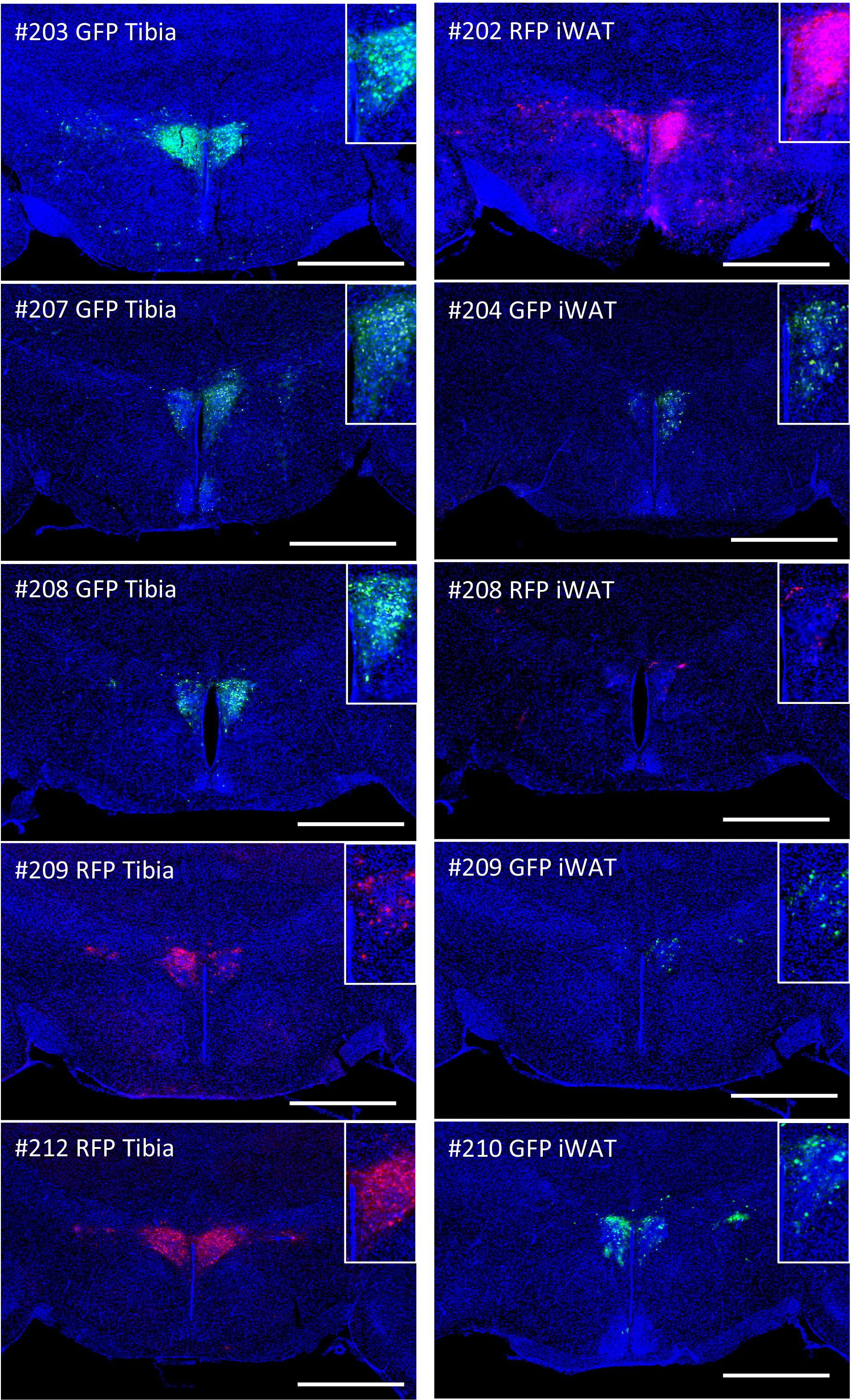

Supplement: Supplemental Figure 1 — Paraventricular hypothalamus from all C3H mice injected with PRV into the tibia or iWAT. Medial portion of the paraventricular hypothalamus from each mouse injected with either PRV-152 (GFP) or PRV-614 (RFP) and the site of injection, tibia or iWAT. Scale bar: 1 mm. [file Image_1.TIF]

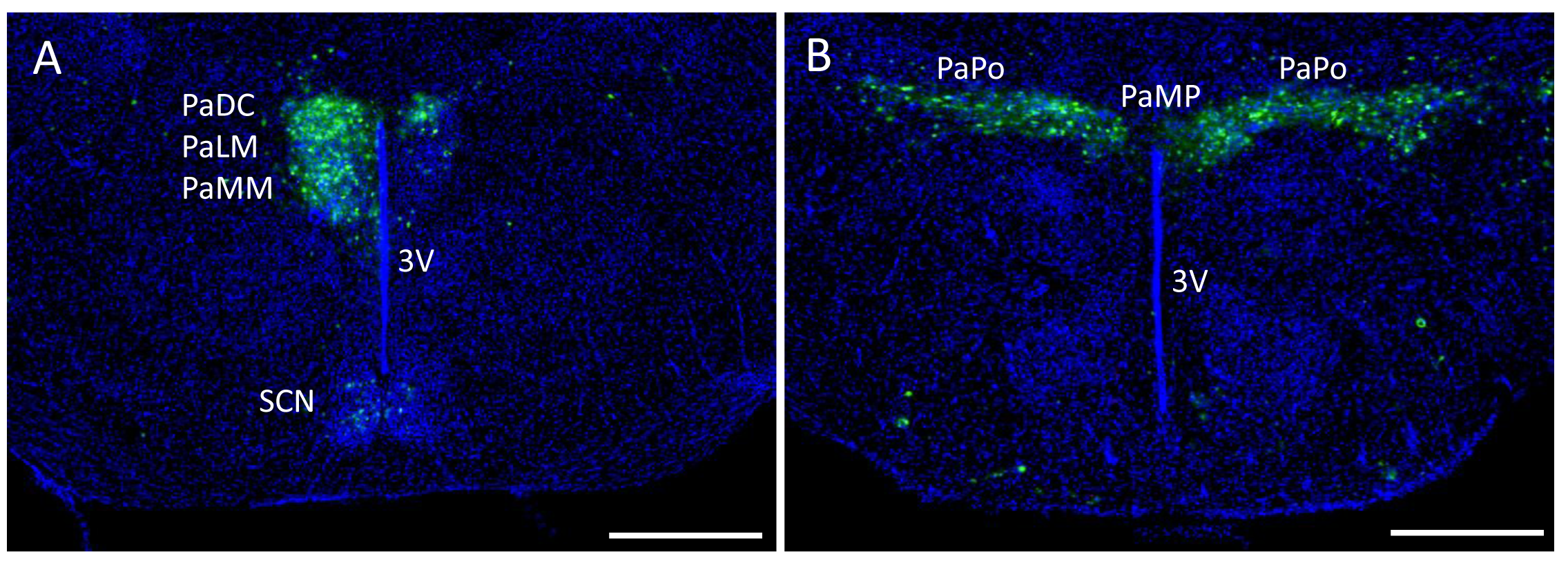

Supplement: Supplemental Figure 2 — PRV infection from bone marrow/BMAT traces to various parts within paraventricular hypothalamus. PRV-152 (GFP) was injected into the tibia and the following sites showed PRV infection: (A) Suprachiasmatic nucleus (SCN) and paraventricular hypothalamic nuclei: dorsal cap (PaDC), lateral magnocellular part (PaLM), and medial magnocellular part (PaMM); (B) Posterior paraventricular hypothalamic nuclei: posterior part (PaMP) and medial parvicellular part (PaPo). [file Image_2.TIF]

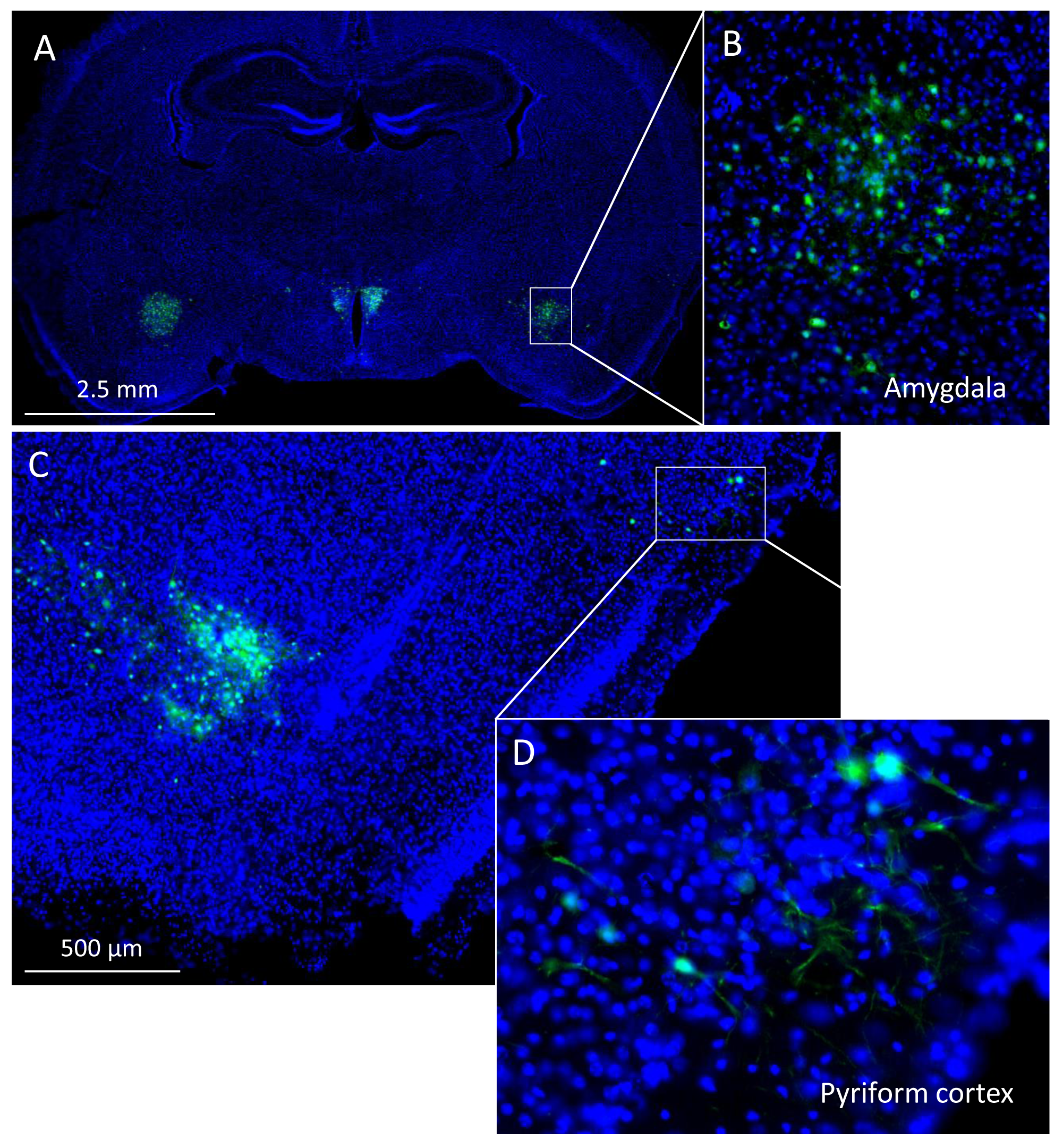

Supplement: Supplemental Figure 3 — Amygdala and pyriform cortex traced from tibia. (A) Overview of brain slice. (B) Amygdala. (C) Overview of pyriform cortex relative to amygdala. (D) Pyriform cortex. [file Image_3.TIF]

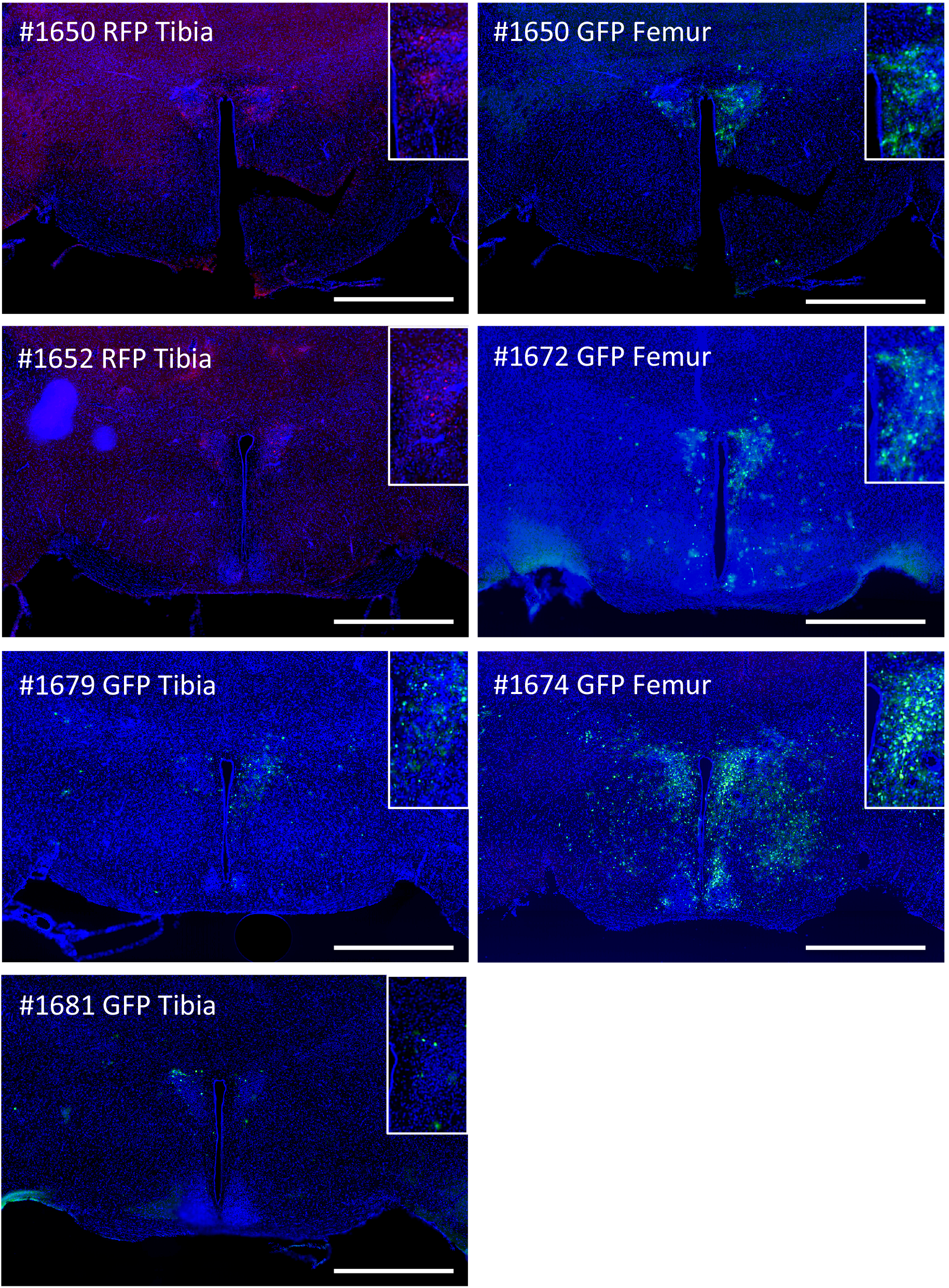

Supplement: Supplemental Figure 4 — Paraventricular hypothalamus from all B6 mice injected with PRV into the tibia or iWAT. Medial portion of the paraventricular hypothalamus from each mouse injected with either PRV-152 (GFP) or PRV-614 (RFP) and the site of injection, tibia or iWAT. Scale bar: 1 mm. [file Image_4.TIF]
