# Supplementary material for: Shared Autonomic Pathways Connect Bone Marrow and Peripheral Adipose Tissues Across the Central Neuraxis
Source: Front Endocrinol (Lausanne). 2019 Sep 27;10:668. doi: 10.3389/fendo.2019.00668 (PMC6776593; doi:10.3389/fendo.2019.00668)
Supplement: Supplemental Table 1 — Antibodies used for immunostaining. [file Table_1.DOCX]

| Tibia |  |  |  |  |
| --- | --- | --- | --- | --- |
| Primary Antibody  (Vendor, Cat. No) | Dilution | Secondary Antibody  (Vendor, Cat. No) | Fluorophore | Dilution |
| Anti-Tyrosine Hydroxylase  (EMD Millipore, Germany, AB152) | 1:1000 | Donkey Anti-Rabbit  (Jackson IR, USA, 711-546-152) | AF488 | 1:200 |
| Anti-Perilipin  (Progen Biotechnik, Germany, GP29) | 1:400 | Donkey Anti-Guinea Pig  (Jackson IR, USA, 706-165-148) | Cy3 | 1:200 |
| Spinal Cord |  |  |  |  |
| Primary Antibody (Vendor, Cat. No) | Dilution | Secondary Antibody | Fluorophore | Dilution |
| Anti-GFP  (Abcam, UK, ab13970) | 1:1000 | Donkey Anti-Chicken  (Jackson IR, USA, 703-545-155) | AF488 | 1:500 |
| Anti-RFP  (Abcam, UK, ab62341) | 1:500 | Donkey Anti-Rabbit  (Jackson IR, USA, 711-585-152) | AF594 | 1:500 |
| Brain |  |  |  |  |
| Primary Antibody (Vendor, Cat. No) | Dilution | Secondary Antibody | Fluorophore | Dilution |
| Anti-GFP  (Abcam, UK, ab13970) | 1:1000 | Donkey Anti-Chicken  (Jackson IR, USA, 703-545-155) | AF488 | 1:500 |
| Anti-RFP  (Abcam, UK, ab62341) | 1:500 | Donkey Anti-Rabbit  (Jackson IR, USA, 711-585-152) | AF594 | 1:500 |
| Anti-MCherry  (SICGEN, Portugal, AB0040-200) | 1:500 | Donkey Anti-Goat (Jackson IR, USA, 705-587-003) | AF594 | 1:500 |
| Anti-Tyrosine Hydroxylase  (EMD Millipore, Germany, AB152) | 1:1000 | Donkey Anti-Rabbit (Jackson IR, USA, 711-605-152) | AF647 | 1:500 |

**Supplemental Table 1. Antibodies used for immunostaining.**
